# Identification of selective hepatitis delta virus ribozyme inhibitors by high-throughput screening of small molecule libraries

**DOI:** 10.1016/j.jhepr.2022.100652

**Published:** 2022-12-17

**Authors:** Eirini D. Tseligka, Stéphanie Conzelmann, Yves Cambet, Tifany Schaer, Francesco Negro, Sophie Clément

**Affiliations:** 1 Department of Pathology and Immunology, University of Geneva, Switzerland; 2 READS Unit, Faculty of Medicine, University of Geneva, Geneva, Switzerland; 3 Clinical Pathology, Geneva University Hospital, Geneva, Switzerland; 4 Gastroenterology and Hepatology, Geneva University Hospital, Geneva, Switzerland

**Keywords:** High-Throughput Screening, Hepatitis Delta Virus, HDV Ribozyme, 8-azaguanine, antiviral drug, CC_50_, half maximal cytotoxic concentration, HBsAg, HBV surface antigen, HBV, hepatitis B virus, HDAC, histone deacetylase, HDV, hepatitis delta virus, HTS, high-throughput screening, IC_50_, half maximal inhibitory concentration

## Abstract

**Background & Aims:**

Chronic hepatitis delta is the most severe form of chronic viral hepatitis and is associated with faster progression towards cirrhosis, liver decompensation, and hepatocellular carcinoma. Hepatitis delta virus (HDV)’s tight dependency on hepatitis B virus and the host cell machinery for its life cycle limits the development of direct-acting antivirals. Thus, we aimed to identify compounds that could block HDV replication by targeting its antigenomic ribozyme.

**Methods:**

We generated stable Huh7 human hepatoma cells expressing a reporter gene (Gaussia luciferase) either downstream (Gluc-2xRz) or upstream (2xRz-Gluc) of two HDV antigenomic ribozyme sequences. We performed high-throughput screening of three small molecule libraries. The secreted luciferase was measured as a readout of ribozyme inhibition upon addition of the molecules. Each plate was considered valid when the Z factor was >0.4. Specificity and toxicity evaluations were performed for the hits with a Z-score >5 and half-maximal inhibitory concentration was calculated by performing a dose-response experiment.

**Results:**

A dose-dependent induction of luciferase expression was detected in Gluc-2xRz-transfected cells incubated with the antisense morpholino, suggesting that the catalytic activity of the ribozyme cloned downstream of the reporter gene was efficiently inhibited. Among the 6,644 compounds screened, we identified four compounds that showed a specific inhibitory effect on the HDV antigenomic ribozyme in Gluc-2xRz cells, *i.e*. three histone deacetylase inhibitors and the purine analogue 8-azaguanine. The latter also significantly decreased HDV replication (by 40%) in differentiated HepaRG cells six days post infection.

**Conclusion:**

Using a novel cell culture model, we identified four small molecules active against the antigenomic HDV ribozyme. These results may provide insights into the structural requirements of molecules designed for the potent and specific inhibition of HDV replication.

**Impact and implications:**

Chronic hepatitis delta is the most severe form of chronic viral hepatitis and is associated with faster progression towards cirrhosis, liver decompensation, and the development of hepatocellular carcinoma. Despite the current development of several new compounds, there is still a need for efficient antiviral treatments specifically targeting hepatitis delta virus (HDV). This work describes a novel cell culture model that allows for the high-throughput screening of compounds able to inhibit HDV ribozymes. We identified four small molecules active against the antigenomic HDV ribozyme (the ribozyme involved in the early step of HDV replication), with the strongest activity shown by 8-azaguanine, a purine analogue. Our data may provide insights into the structural requirements of molecules designed to inhibit HDV.

## Highlights

• HDV infection, for which there are limited treatment options, is the most aggressive form of chronic viral hepatitis.
• We developed a novel cell-based high throughput assay targeting the HDV ribozyme.
• The identified compounds could guide the development of a new class of antivirals that target the HDV ribozyme.

## Graphical abstract

**Figure:**
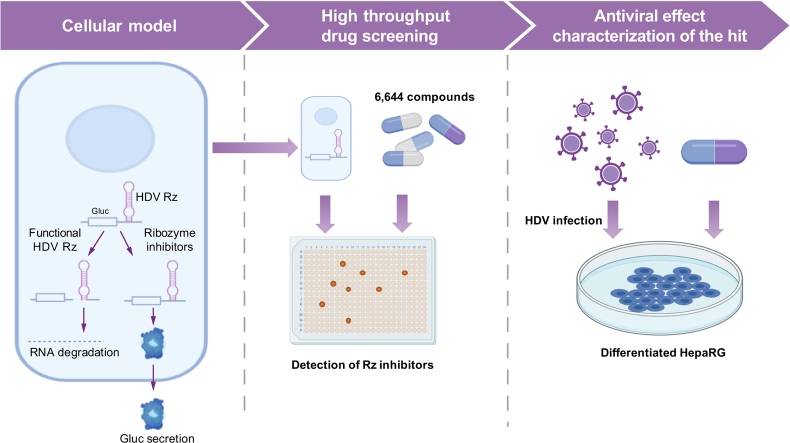

## Introduction

Hepatitis delta virus (HDV) is a small virus (35-37 nm), with a single-stranded circular RNA genome (∼1,700 nucleotides) of negative polarity. HDV is a defective virus as it does not code for its own envelope proteins and thus depends on hepatitis B virus (HBV) surface antigen (HBsAg) expression in the same cell to complete the assembly of viral particles.^1^ HDV infection therefore only occurs as either coinfection with HBV or superinfection of an HBV carrier. HDV affects ∼5% of the global HBV-infected reservoir, and is associated with a faster progression towards cirrhosis, liver decompensation, and liver-related mortality compared to HBV monoinfection.^2^ We recently reported that patients with HDV infection are at a significantly higher risk of developing hepatocellular carcinoma compared to HBV-monoinfected patients.^3^

Until recently, the only available therapeutic option to treat HDV (although not officially approved for this indication) was interferon-alpha in its pegylated formulation, which is poorly tolerated, has many contraindications and lacks sufficient effectiveness. In July 2020, the viral entry inhibitor bulevirtide was conditionally approved by the EMA for the treatment of patients with chronic hepatitis D and compensated liver disease.^4^ Two additional anti-HDV compounds, the prenylation inhibitor lonafarnib and the nucleic acid polymer REP-2139 have shown encouraging response rates, but additional data may be warranted prior to their approval by regulatory authorities.^5^ In addition, while HBV vaccination protects against both viruses, all attempts to develop a vaccine preventing HDV superinfection of HBV carriers have failed so far.^6^ There is thus an urgent need to develop additional, efficient antiviral therapies against HDV.

The replication of HDV RNA follows a rolling-circle mechanism^7^ in the nucleus of hepatocytes and is completely independent of HBV replication. Furthermore, HDV does not encode its own RNA-dependent RNA polymerase, but hijacks the host DNA-dependent RNA polymerases II,^8^^,^^9^ I and III.^10^ This tight dependency of HDV on the host cell machinery for its life cycle poses evident challenges to the development of drugs specifically targeting the HDV life cycle. However, both the genomic and the antigenomic strands of HDV possess a unique, specific RNA motif capable of self-cleavage activity (ribozyme) that intervenes at different stages of the viral life cycle.^11^ In fact, once in hepatocyte nuclei, the circular genomic RNA is transcribed either into (i) a short 800 nucleotide-long antigenomic mRNA that undergoes capping and polyadenylation prior to translation into HDAg (the delta antigen),^9^ or (ii) concatemeric antigenomic RNAs that undergo self-cleavage to unit-length antigenomic transcripts via the HDV antigenomic ribozyme.^12^^,^^13^ The antigenomic HDV monomers are then ligated by the cellular machinery into circular RNAs^14^ that undergo the same rolling-circle transcription mechanism, leading to concatemeric genomic RNAs, further self-cleaved into unit-length genomic transcripts by the HDV genomic ribozyme. The latter is then ligated to yield circular genomic RNAs^14^ that are packaged into HDV particles. These two ribozyme activities are the only enzymatic activities performed by HDV itself; thus, they may be suitable targets for direct-acting antivirals. A previous study demonstrated the effective inhibition (up to 95%) of the autocatalytic activity of HDV genomic Rz *in vitro* by aminoglycosides; however, none of these molecules have proven to be active *in vivo*.^15^ More recently, an elegant cell culture system enabling the identification of inhibitors of ribozyme activity via high-throughput screening (HTS) of chemical libraries has been described.^16^ This system is based on the incorporation of ribozyme sequences into the transcription unit of a reporter gene. During mRNA transcription, spontaneous self-cleavage by the ribozyme should lead to the degradation of the mRNA, thus abolishing expression of the reporter gene. Administration of inhibitors targeting the ribozyme should block mRNA degradation and thus restore protein expression. The authors applied this approach to a specific human parasite (Schistosoma mansoni) ribozyme named N79 and successfully identified 15 compounds able to inhibit its activity.

Herein, we applied this approach to the screening of compounds inhibiting the HDV antigenomic ribozyme. To this aim, we generated stable Huh-7 hepatoma cells expressing secreted Gaussia luciferase (Gluc) under the control of the HDV antigenomic ribozyme. By performing HTS of three commercially available drug libraries, we identified four small molecules active against HDV. In particular, 8-azaguanine, a purine analogue, demonstrated the most effective inhibition of the HDV ribozyme.

## Materials and methods

### Cells

Human hepatocyte carcinoma cells (Huh7) were cultured in DMEM supplemented with 10% FBS, 1% L-glutamine, 1% penicillin-streptomycin (all from Gibco, Thermo Fisher Scientific). HepNB2.7 (provided by Prof. Stephan Urban, University Hospital Heidelberg, Germany) were cultured in the above medium supplemented with 5 μg/ml of puromycin (ant-pr-1, InvivoGen). The human liver progenitor HepaRG cells (provided by Dr. Julie Lucifora, INSERM, Université de Lyon, France) were cultured and differentiated as previously described.^17^ Cultures were maintained at 37 °C in a humidified atmosphere of 5% CO_2_.

Cell viability was assessed using the MTT assay (Sigma-Aldrich, In Vitro Toxicology Assay Kit, TOX1-1 KT) on differentiated HepaRG, Huh7 (4.5x10^3^ cells/well) and HepNB2.7 (10x10^3^ cells/well) cells plated in 96 well plates 16 h prior to treatment for 6 days with different doses of the selected compounds.

### Expression vectors

**Fig. 1:**
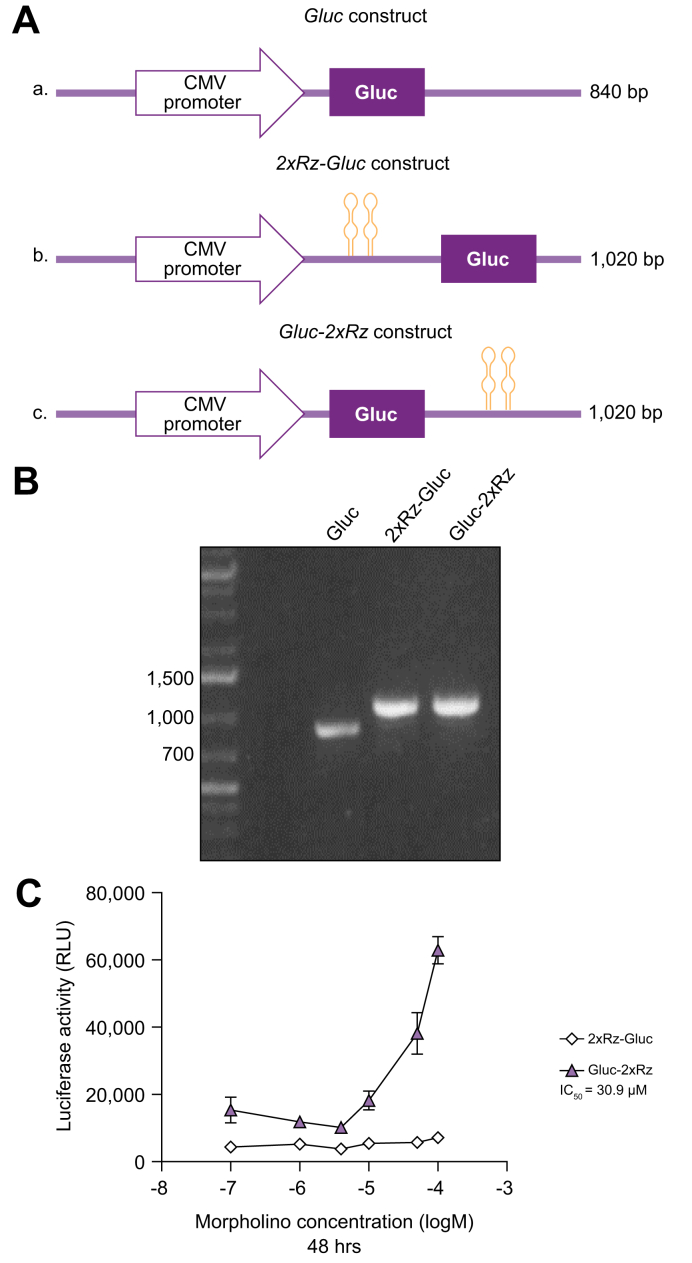
Cell-based assay development and validation. (A) Schematic representation of the constructs used for the generation of stable cell lines: a) Gluc, b) 2xRz-Gluc, and c) Gluc-2xRz constructs. (B) DNA from stably transfected Huh7 cells was extracted and PCR amplification of the inserts was performed using the primers CMV-IE and M13 -Fw. The agarose gel demonstrates the efficient transfection of Huh7 cells and the sizes of the Gluc (840 bp), 2xRz-Gluc (1,020 bp) and Gluc-2xRz (1,020 bp) constructs. (C) Dose-response experiments in 2xRz -Gluc and Gluc- 2xRz cells treated with increasing concentrations of antisense morpholino. No effect in the luciferase induction was observed in 2xRz-Gluc-transfected cells treated with the antisense morpholino, whereas a dose-mediated induction of luciferase expression was detected in Gluc-2xRz-transfected cells incubated with the antisense morpholino (IC_50_ = 30.9 μM). RLU, relative luminescent unit.

The gene of secreted Gluc was cloned between the BamH1 and HindIII (New England Biolabs) restriction sites in pBApo-CMV Pur (GenScript Biotech Corporation) either downstream or upstream of two HDV antigenomic ribozyme sequences, as previously described^16^ (Fig. 1A). All the sequences were verified with Sanger sequencing (Fasteris-DNA sequencing service, Switzerland).

### Generation of stable cell lines expressing the HDV ribozyme, DNA extraction and PCR

Vectors were linearized with EcoRI (New England Biolabs) and dephosphorylated with the FastAP Thermosensitive Alkaline Phosphatase (ThermoFischer) to avoid re-ligation of the linearized vector according to the manufacturer’s instructions. The linearized product was purified using a purification column (ReliaPrep™ DNA Clean-up and Concentration System, Promega).

Huh7 cells were plated until they reached a confluency of 80% and were transfected with 2 μg of linearized vectors using the jetPRIME^R^ transfection reagent (Polyplus) according to the manufacturer’s instructions. Twenty hours post transfection, the cells were subjected to puromycin selection (Invivogen, Labforce 4 μg/ml). Stably transfected Huh7 cells were maintained in the selection medium.

DNA from stably transfected Huh7 cells was extracted (NucleoSpin Tissue, Macherey-Nagel) and PCR amplification was performed using primers (CMV-IE 5’CGCAAATGGGCGGTAGGCGTG3’ and M13 5’TGTAAAACGACGGCCAGT3’) targeting the multiple cloning site of the pBApo-CMV Pur vector and the platinum Taq DNA polymerase (NEB, #M0267). PCR products were purified as previously described^18^ and sequenced with the M13 reverse primer.

### Compound libraries and reagents

Three libraries were screened: i) APExBio (Apexbt) (1,971 approved drugs targeting more than 20 signaling pathways, *i.e.* DNA damage/repair, cell cycle, angiogenesis, *etc.*), ii) Prestwick (Prestwick Chemical Libraries) (1,280 small molecules, 95% of which are approved drugs with known bioavailability and safety in humans), and iii) Enamine (SIA Enamine) (3,393 compounds with antiviral activity). All the compounds used for the screening validation and the dose-response experiments were purchased from MedChemExpress. DMSO was used as a vehicle.

The specific morpholino targeting the HDV antigenomic ribozyme (TGGCGATGCCATGCCGACCC) was purchased from GeneTools (USA) and was transfected into the cells using the jetPRIME^R^ transfection reagent according to the manufacturer’s instructions. Morpholinos are antisense oligonucleotides that bind to specific target sites and can be used to block access to RNA. They are commonly used to block mRNA translation, pre-mRNA splicing, miRNAs or their targets, and ribozyme activity.

The secreted luciferase was quantified in the cell supernatant using the Secrete-Pair™ Gaussia Luciferase Assay (Labomics) according to the manufacturer’s instructions. The luminescence levels were quantified with the FDSSμCELL reader (Functional Drug Screening System, Hamamatsu).

### HTS: assay development and validation

Gluc-2xRz cells (5x10^3^cells/well) and Gluc (1x10^3^cells/well) were plated in 384 well plates (Corning) and incubated with the compounds (10 μM resuspended in DMSO) of each library for 48 h. Gluc cells were included as a negative control and all the compounds with non-specific effects in these cells were excluded from our analysis.

Dose-response effects (30 μM – 60 nM*)* and toxicity evaluation of the hits with Z-scores >5 were calculated during the HTS assay validation experiments. All data were generated from two independent experiments.

### *In vitro* transcription and ribozyme self-cleavage

The plasmid pET-20b(+) (GenScript Biotech Corporation) containing the HDV ribozyme sequence under a T7 promoter was linearized using the restriction enzyme *Nae*I (new England Biolabs). After DNA extraction using Phenol:chloroform:isoamyl alcohol (25:24:1), the ribozyme RNA was *in vitro* transcribed using the MEGAshortscript™ kit (Invitrogen) and treated with either morpholino (10 μM) or 8-azaguanine (10 and 100 μM). For experiments of incorporation into the ribozyme, transcription was performed by substituting GTP by 8-azaguanosine-5’-triphosphate (custom synthesis carried out by Jena Bioscience GmbH, Germany) at several relative proportions. RNA transcripts were purified using the NucleoSpin RNA II kit (Macherey-Nagel), denatured at 95 °C for 3 min in 50 mM Tris-HCl (pH 7.5) and slowly cooled to room temperature for 15 min. The cleavage reaction was induced by adding 10 mM MgCl_2_ and incubating for 1 h at 37 °C. RNA fragment sizes and ribozyme cleavage activity were assessed using the bioanalyser (Agilent RNA 6000 pico kit).

### Viral stocks and infection

HDV particles were produced by cotransfecting Huh7 cells with a trimer HDV-1 prototype replication-competent plasmid (pSVLD3) and an HBsAg-encoding plasmid (pT7HB2.7), before being tittered as previously described.^19^

Differentiated HepaRG cells, HepNB2.7 (10x10^3^ cells/well) cells were infected with 50 viral genome equivalents/cell of HDV using 4% polyethylene glycol overnight as previously described.^19^

### HDV replication quantification

For HDV quantification, total intracellular RNA was extracted using the Nucleospin RNA Kit (Macherey-Nagel AG). cDNA was synthesized from 100 ng total RNA using Superscript II and random hexamer primers (Roche Diagnosis). Quantification of HDV was performed by quantitative reverse-transcription PCR as described,^19^ using the primers described by Scholtes & colleagues^20^ and EEF1A1 as a housekeeping gene.

### 8-azaguanine HDV inhibition assay

8-azaguanine (MedChemExpress) was serially diluted and added to the cell culture media once at 16 h post HDV infection or twice (16 h and 3 days post infection). Three and six days post infection, RNA was extracted and HDV RNA level was monitored by quantitative reverse-transcription PCR.

### Statistical analyses

Statistical analyses of the HTS were performed using an excel macro based on Genedata and ActivityBase softwares (IDBS) calculating the Z-score (the number of standard deviations from the mean score) of each compound: Z-score = (relative luminescent unit of each compound – relative luminescent unit of control)/standard deviation of control. Gluc-2xRz cells were used as controls. The outliers were excluded from our data set according to the Thompson Tau test. Each plate with Z factor >0.4 was considered valid.^21^ The normalized percentage of inhibition was calculated as (data – mean DMSO)/(mean morpholino – mean DMSO).

Values were expressed as mean (±SEM). Experiments were performed at least in biological duplicates. *T*-tests were performed and the half maximal inhibitory (IC_50_), and cytotoxic (CC_50_) concentrations were determined using GraphPad Prism 9.1.0 software.

## Results

### Assay development and validation

We first generated stable Huh7 human hepatoma cells expressing the antigenomic HDV ribozyme, either upstream (Huh7-2xRz-Gluc) or downstream (Huh7-Gluc-2xRz) of the Gluc reporter gene (Fig. 1A). The correct insertion of the cassettes in the expression vectors was confirmed by PCR amplification (Fig. 1B).

To validate the experimental strategy, we assessed the induction of luciferase expression in the presence of an antisense morpholino oligonucleotide specifically designed to hybridize with the cleavage site of the HDV ribozyme and thus to block its self-cleavage activity, as described.^16^ As shown in Fig. 1C and Fig. S1, the basal luciferase activity in 2xRz-Gluc-transfected cells remained unaffected by the antisense morpholino targeting the catalytic activity of the ribozyme inserted upstream of the reporter gene. On the contrary, a dose-dependent induction of the luciferase expression was detected in Gluc-2xRz-transfected cells incubated with the antisense morpholino for 24, 48, or 72 h, therefore implying an efficient inhibition of the HDV antigenomic self-cleavage activity when the ribozyme sequences were inserted downstream of the Gluc gene (IC_50_ = 30.9 μM after 48 h), thus making this cell line a suitable model to screen compounds for their potential antiviral activity.

### HTS for identifying HDV Rz inhibitors and hit validation

**Fig. 2:**
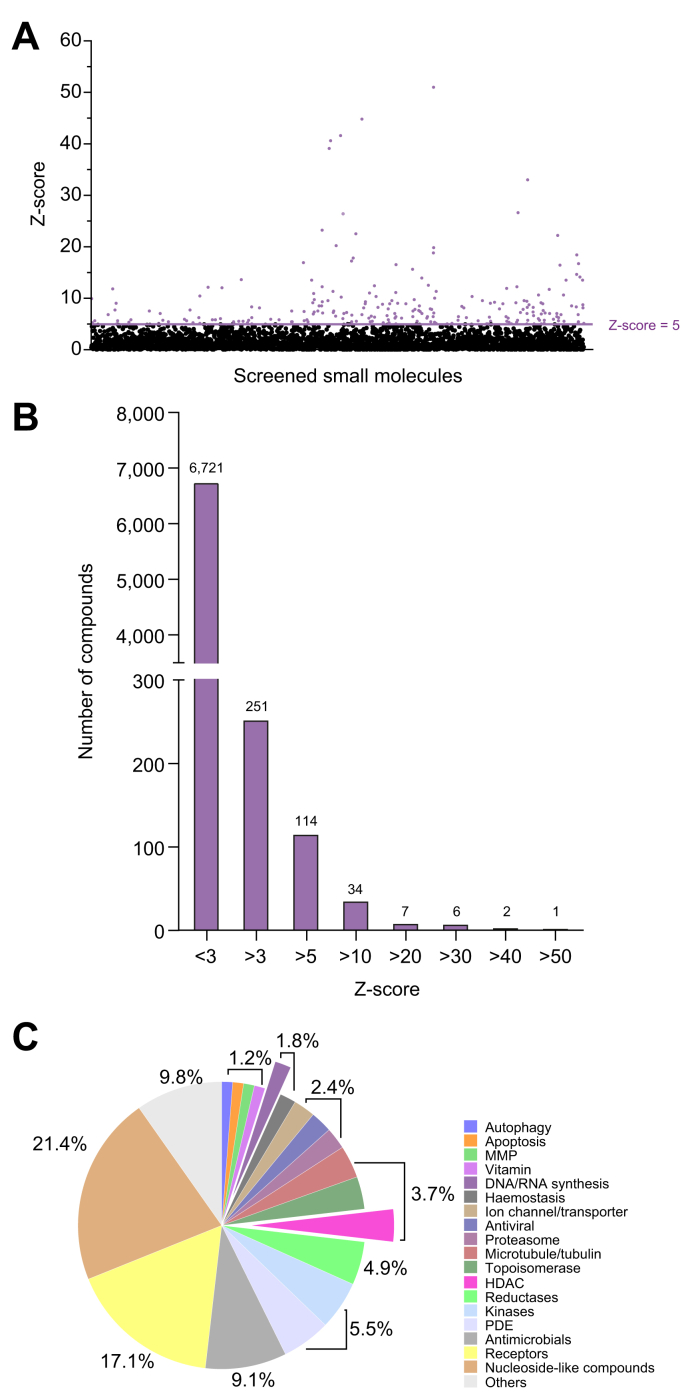
High-throughput screening of small molecule libraries in Gluc-2xRz cells. (A) Z-score values calculated for each compound (10 μM) of the libraries. The luciferase activity was measured 48 h after the treatment of Gluc-2xRz cells with the compounds (n = 2). Hits with a Z-score >5 are highlighted in red. (B) Graph representing the number of compounds based on their Z-score values. Positive hits with Z-score >5 were selected for further analysis and data validation. (C) Pie chart demonstrating the distribution of the hits with Z-score >5 according to the target drugs. The exploded slices represent the group of compounds interfering with DNA/RNA synthesis, including 8-azaguanine (red) and the inhibitors of HDAC (magenta), respectively. HDAC, histone deacetylase; MMP, matrix metalloproteinases; PDE, phosphodiesterase.

We performed a HTS on Gluc-2xRz cells, including Gluc-2xRz cells treated with 100 μM of the antisense morpholino as a positive control. As an additional control, we used Huh7 cells expressing Gluc without ribozyme sequences. We screened all three libraries (for a total of 6,644 compounds) at 10 μM on Gluc-2xRz cells plated at 5,000 cells/well in 384-well plates. A representative heat-map of the positive hits from the ApexBio FDA-approved library demonstrates the Z-score values for the compounds (Fig. 2A). From the initial single-dose screening, we selected 164 compounds with a Z-score >5 for further validation, as they represent compounds that are statistically different (>5 SD) from the control population of our assay (Gluc-2xRz) (Fig. 2B). All the compounds were classified according to the information provided for each screened library (Table S1). Among them, the most represented were receptor inhibitors (17.1%) or nucleoside-like compounds that originated from the Enamine antiviral library (21.4%) (Fig. 2C).

**Fig. 3:**
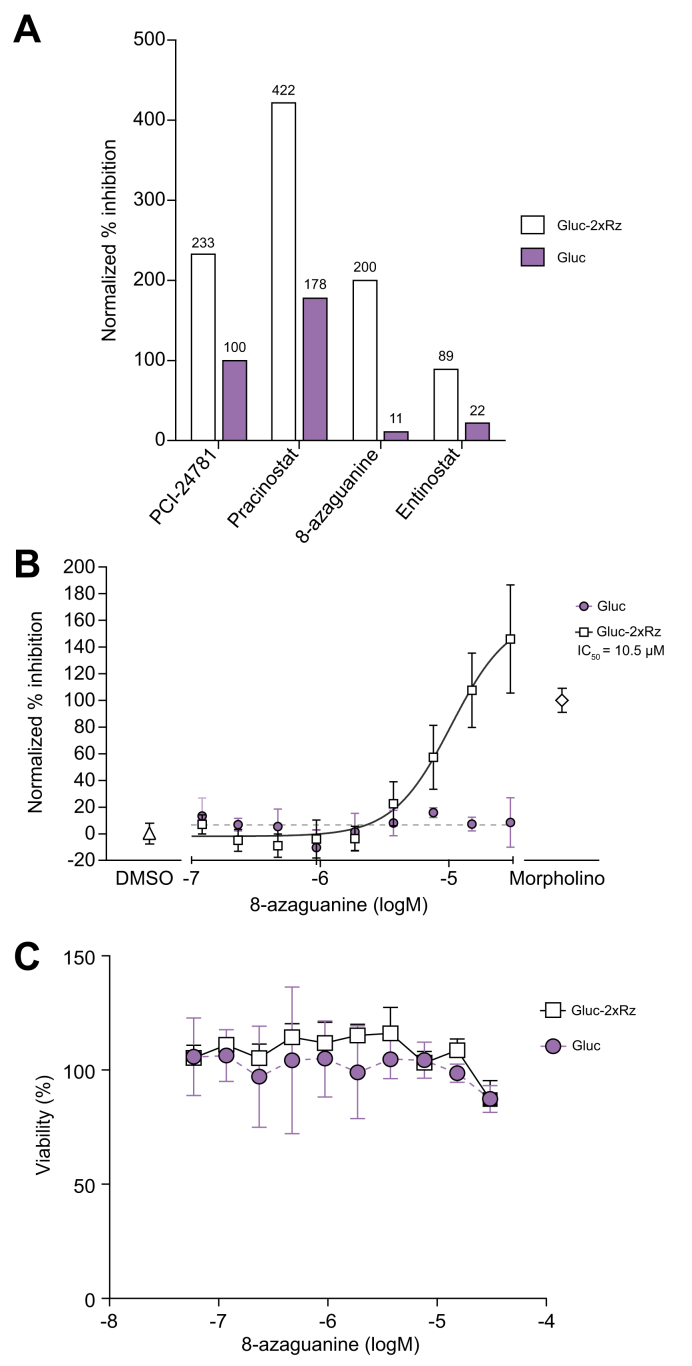
Hit validation on Gluc-2xRz and Gluc cells. (A) Among the 131 compounds that were further validated at 10 μM only four of them specifically targeted HDV antigenomic Rz and had lower or almost no effect in the Gluc cells as demonstrated by the Z-score values. (B) IC_50_ of 8-azaguanine in Gluc-2xRz and Gluc cells. (C) Toxicity evaluation of 8-azaguanine by MTT assay. IC_50_, half-maximal inhibitory concentration. Data are presented as the mean (± SEM) of triplicate.

Among the 164 compounds selected from the original screening, we were able to purchase 131 compounds from the same supplier to build a home-made library. The other 30 compounds were not available because their synthesis had been discontinued. Validation experiments of the 131 compounds diluted at 10 μM were performed on the Gluc-2xRz and Gluc cells to ensure the specific induction of Gluc due to the inhibition of the HDV antigenomic ribozyme (Fig. 3). Four compounds from the ApexBio FDA-approved library – three histone deacetylase (HDAC) inhibitors (PC1-24781, pracinostat and entinostat) and a purine analogue (8-azaguanine) showed a specific inhibitory effect on the HDV antigenomic ribozyme, as indicated by the percentage of inhibition in Gluc-2xRz cells at a concentration of 10 μM, which was at least twofold higher than in Gluc cells (Fig. 3A). From these four molecules, 8-azaguanine demonstrated the most specific effect with a Z-score of 18 in the Gluc-2xRz cells and a Z-score of 1 in Gluc cells. The remaining 127 hits were excluded due to non-specific interference with luciferase activity.

Dose-response experiments with the four compounds applied at dilutions ranging from 30 μM to 60 nM further confirmed the specificity of 8-azaguanine, with an IC_50_ of 10.5 μM in the Gluc-2xRz cells and no effect in Gluc cells (Fig. 3B, Fig. S2). No toxicity in either cell line was observed (Fig. 3C).

### Mechanism of inhibitory activity of 8-azaguanine on HDV ribozyme

**Fig. 4:**
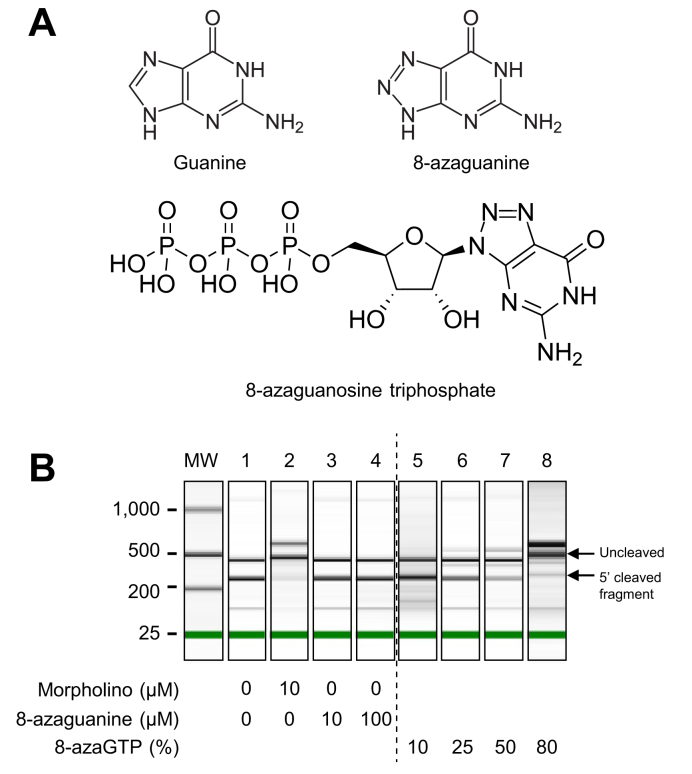
*In vitro* self-cleavage of HDV ribozyme in presence of 8-azaguanine or 8-azaGTP. (A) Chemical structures of guanine, 8-azaguanine and 8-azaguanosine triphosphate (8-azaGTP). (B) In vitro-transcribed ribozyme was incubated with morpholino (10 μM) or 8-azaguanine (10 μM or 100 μM) prior to the cleavage reaction which was induced by adding 10 mM MgCl_2_ (lanes 1-4). For experiments of incorporation into the ribozyme, in vitro transcription was performed by substituting GTP with 8-azaguanosine-5’-triphosphate at 10 to 80% (lanes 5-8) and the cleavage was then induced on the resulting ribozyme in which guanosines were partially replaced by 8-azaguanosines.

Two mechanisms may explain the inhibitory activity of 8-azaguanine: a direct interaction between 8-azaguanine and the ribozyme or its incorporation into the RNA. To test the first hypothesis, we incubated the *in vitro*-transcribed ribozyme with 8-azaguanine. Contrary to the morpholino (Fig. 4B, lane 2), 8-azaguanine did not inhibit the self-cleavage of the ribozyme (Fig. 4, lanes 3-4). To assess whether 8-azaguanine may exert its inhibitory activity after being incorporated into the RNA molecule, we *in vitro* transcribed the ribozyme sequence using 8-azaguanosine-5’-triphosphate (Fig. 4A) instead of GTP at increasing relative proportions. As shown in Fig. 4B (lanes 5-8), substitution of guanosines by 8-azaguanosine induced an inhibition of ribozyme self-cleavage in a dose-dependent manner.

### Inhibitory activity of 8-azaguanine on HDV replication

We further assessed the antiviral potency of 8-azaguanine in (i) HDV-infected, differentiated HepaRG cells,^19^ and (ii) HepNB2.7 cells that stably express the HBV envelope proteins and therefore recapitulate the full HDV life cycle.^22^

**Fig. 5:**
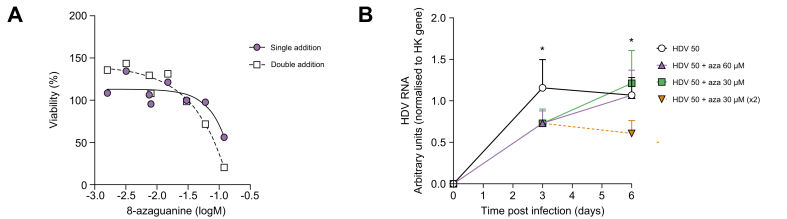
Toxicity and antiviral effect of 8-azaguanine on differentiated HepaRG cells. (A) Viability of differentiated HepaRG cells treated with increasing concentrations of 8-azaguanine was assessed at day 6 using the MTT assay. The drug was applied either once (at day 0, solid line) or twice (at day 0 and 3, dotted line). No cytotoxicity was detected at 60 μM (single addition) and 30 μM (single and double addition). (B) Differentiated HepaRG cells were infected with 50 vge/cell of HDV and treated with 8-azaguanine at 30 μM and 60 μM 16 h post-infection. For the concentration of 30 μM, 8-azaguanine was added either once (16 h post infection, grey solid line) or twice (second treatment at 3 days, grey dotted line). Levels of intracellular HDV RNA were assessed by RT-qPCR at 3- and 6-days post infection. Data are expressed as the mean ±SEM of three independent experiments. ^∗^*p* ≤0.05 compared to untreated HDV infected controls. RT-qPCR, reverse-transcription quantitative PCR; vge, viral genome equivalents.

We performed a cytotoxicity assay to define non-toxic doses of 8-azaguanine in both cellular models. The CC_50_ was 125.9 μM in differentiated HepaRG (Fig. 5A). Even after only three days of treatment, 8-azaguanine was highly cytotoxic in HepNB2.7 cells, with a CC_50_ value of 19.4 μM (Fig. S3).

To determine the antiviral activity of 8-azaguanine, differentiated HepaRG were infected with HDV at 50 viral genome equivalents/cell and subsequently treated with either 60 or 30 μM (single or double dose). HDV replication was assessed 3- and 6-days post infection by measuring the amount of HDV RNA in cell extract by reverse-transcription PCR. As shown in Fig. 5B, both concentrations induced a reduction of HDV replication at day 3, which was only maintained at day 6 when 8-azaguanine was applied twice (16 h and 3 days post infection) at 30 μM. It is noteworthy that, in these experimental conditions, 8-azaguanine was not cytotoxic in the context of infection (Fig. S4).

Given that HepNB2.7 cells were more sensitive to 8-azaguanine, we treated them with lower concentrations (7.5 and 3.2 μM), which failed to show any inhibitory effect on HDV replication (Fig. S3).

## Discussion

Chronic HDV infection is considered the most aggressive form of chronic viral hepatitis and is associated with faster progression towards end-stage liver diseases than HBV monoinfection. To date, the drug development pipeline remains limited due to the close dependency of HDV on the host cell machinery for its life cycle, thus highlighting the need to develop novel antiviral therapies. However, not all antiviral approaches have been duly investigated. For example, the ability of short-interfering RNAs, which are currently under clinical development for HBV, to block HDV replication warrants investigation.^23^ Similarly, the ribozyme activities intrinsic to HDV have not received sufficient attention as antiviral targets. Thus, we have established and validated a sensitive cell-based assay suitable for HTS of small molecules capable of inhibiting HDV’s antigenomic ribozyme activity. To our knowledge, this is the first study that aims to repurpose FDA-approved or antiviral compounds against HDV ribozymes. We identified four compounds, including three HDAC inhibitors and a purine analogue, which specifically blocked the HDV antigenomic ribozyme. More importantly, we could confirm the anti-HDV activity of the purine analogue 8-azaguanine in HDV-infected differentiated HepaRG cells.

Our model has several advantages for the screening of HDV ribozyme inhibitors. First, it is based on a simple and clear-cut readout and a single target rather than the use of a fully infectious system, thus simplifying data interpretation. One of the main challenges during drug library screening is the presence of hit compounds generating non-specific assay interference. Here, we developed a gain-of-signal luciferase assay, thereby minimizing false positive hits that are cytotoxic. Nevertheless, considering previous studies showing that (i) the amount of HDV RNA in an *in vitro*-infected cell can reach approximately 100,000 copies after 5-6 days of infection;^24^ (ii) the HDV antigenomic RNA is estimated to be 5x to 20x less abundant than the genomic RNA;^25^ and (iii) foreign promoters, such as a CMV promoter, are thought to express mRNA at levels >100,000 copies per cell,^26^ we can roughly estimate that we may have faced up to a 10-fold excess of antigenomic ribozyme RNA compared to naturally infected hepatocytes. This supraphysiologic level of target RNA may have set a high threshold to identify inhibitors, reducing the sensitivity of the model but at the same time increasing its specificity. Moreover, the first round of screening identified hits that, in the end, showed non-specific effects on luciferase. It is noteworthy that some of these non-specific compounds belonged to the proteasome inhibitor family, suggesting that these molecules induced an increased Gluc signal by inhibiting its degradation by the proteasome. During the validation process, we observed that ribozyme self-cleavage was different depending on whether Gluc was cloned upstream or downstream, in line with a previous study showing that the nature of the sequence surrounding the cleavage site of the HDV ribozyme may influence the formation of active ribozymes.^27^ One explanation for this result could lie in the fact that, when the ribozyme is cloned downstream of the Gluc cassette, ribozyme cleavage removes the polyA tail of the mRNA which becomes unstable.

Among the compounds displaying an inhibitory effect on ribozyme activity, we identified three compounds belonging to the HDAC inhibitor family. It has previously been shown that HDAC inhibitors can represent new therapeutic tools against viral infections including HIV and CMV, as they can reactivate the latent virus present in infected cells and induce viral clearance from cellular reservoirs.^28^ However, as far as ribozyme activity is concerned, no data on a potential mechanism of action of these enzymes is available.

The most potent anti-HDV ribozyme molecule identified in this study was 8-azaguanine, a purine analogue with antineoplastic activity that has been previously used for the treatment of acute leukemia.^29^ Acyclic analogues of 8-azaguanine have previously been shown to exhibit an antiviral activity against herpes simplex virus 1 and 2, CMV, varicella-zoster virus, HIV-1 and 2.^30^ It has been reported that 8-azaguanine, after its conversion into a nucleoside by the hypoxanthine phosphoribosyltransferase can compete with purines for incorporation into tRNA catalyzed by the enzyme tRNA-purine transglycosylase.^31^

The structures of the HDV ribozymes consist of five paired (P1, P1.1, P2, P3 and P4) segments that form a nested double pseudoknot.^32^ The ribozyme promotes cleavage of the RNA at the base of the P1 region through a nucleophilic attack by a 2’ hydroxyl group of the -1 nucleotide on the adjacent phosphate.^12^ In our context, it is noteworthy that some nucleotides (and notably guanosine residues) that are part of the ribozyme sequence are highly conserved in all HDV variants and are considered as the “signature” of HDV ribozymes. The significant number of guanosines surrounding the cleavage site of the antigenomic ribozyme and the aforementioned mechanism of action of 8-azaguanine strongly suggest that the latter may be incorporated into the sequence of the antigenomic ribozyme of HDV, therefore interfering with its autocatalytic activity. Our data of *in vitro* transcription showing that the substitution of guanosines by 8-azaguanosine induced an inhibition of ribozyme self-cleavage are strong evidence in favor of this hypothesis and provide some insights on the mechanism of action of 8-azaguanine.

One limitation of these base analogues lies in the fact that they are highly mutagenic, leading to cell death. We observed a high toxicity of 8-azaguanine in HepNB2.7 cells, which are highly proliferative. On the contrary, differentiated non-dividing HepaRG cells showed much better tolerance. This observation is in line with the known antineoplastic activity of 8-azaguanine in animal models.^33^ An alternative mechanism may be the blockade of the initiation of translation and an inhibition of protein synthesis by interfering with the formation of 43S and 80S initiation complexes.^34^ In differentiated HepaRG cells, we did not observe any obvious protein synthesis inhibition (data not shown), suggesting that, with the concentrations applied to the cells, this particular action of the drug may not be relevant. Another limitation was represented by the micromolar concentration required to inhibit the HDV ribozyme. Thus, we want to emphasize the fact that our findings do not identify 8-azaguanine as potential direct-acting antiviral to be applied in the clinical setting. The molecules identified, in particular 8-azaguanine, may however represent the starting point for the *in silico*, structural optimization of further direct-acting antivirals targeting the HDV ribozyme.

In conclusion, we developed a new cellular model that may prove helpful in the discovery of novel compounds that target the ribozyme activity of HDV. These results provide proof-of-concept of the feasibility of anti-ribozyme targeting as an antiviral approach.

## Financial support

This work is supported by the PRD (Projets Recherche & développement) of the Geneva University Hospital, Switzerland, (grant #PRD 4-2020-II_CGR 71344 to EDT). The funders had no role in study design, data collection and analysis, decision to publish, or preparation of the manuscript.

## Authors’ contributions

EDT: funding acquisition, project administration, investigation, data acquisition, validation, data analysis, writing – original draft, writing – review & editing; YC: data acquisition, statistical analysis; StC and TS: data acquisition; FN: conceptualization, funding acquisition, supervision, project administration, writing – review & editing; SoC: conceptualization, funding acquisition, project administration, supervision, writing – original draft, writing – review & editing.

## Data availability statement

The authors confirm that the data supporting the findings of this study are available within the article and Supplementary information. Any additional data are available from the corresponding author F.N. upon reasonable request.

## Conflict of interest

The authors declare no conflicts of interest that pertain to this work.

Please refer to the accompanying ICMJE disclosure forms for further details.
