# Supplementary material for: Identification of selective hepatitis delta virus ribozyme inhibitors by high-throughput screening of small molecule libraries
Source: JHEP Rep. 2022 Dec 17;5(3):100652. doi: 10.1016/j.jhepr.2022.100652 (PMC9871325; doi:10.1016/j.jhepr.2022.100652)
Supplement: Multimedia component 1 [file mmc1.pdf]

Journal of Hepatology, Volume *5*

Supplemental information

Identification of selective hepatitis delta virus ribozyme inhibitors by high-throughput screening of small molecule libraries

Eirini D. Tseligka, Stéphanie Conzelmann, Yves Cambet, Tifany Schaer, Francesco Negro, and Sophie Clément

Identification of selective hepatitis delta virus ribozyme inhibitors by high-throughput screening of small molecule libraries

Eirini D. Tseligka, Stéphanie Conzelmann, Yves Cambet, Tifany Schaer, Francesco Negro, Sophie Clément

Table of contents

Supplementary materials and methods 2
Fig. S1 4
Fig. S2 5
Fig. S3 6
Fig. S4 7
Supplementary references 7

Supplementary materials and methods

Cell lines

Name	Citation	Supplier	Passage no.
HepaRG	(1) (2)	Provided by Julie Lucifora, INSERM, Lyon	Up to 20
HepNB2.7	(3)	Provided by Prof. Stephan Urban, University Hospital Heidelberg, Germany	Up to 20

Sequence based reagents

Name	Sequence	Supplier
Morpholino	TGGCGATGCCATGCCGACCC	GeneTools (USA)
Primer CMV-IE	5'CGCAAATGGGCGGTAGGCGTG3'	Microsynth
Primer M13	5'TGTAAAACGACGGCCAGT3'	Microsynth
Primers EEF1A1	Fwd : AGCAAAAATGACCCACCAATG<br>Reverse : GGCCTGGATGGTTCAGGATA	Microsynth
Primers HDV	Fwd : CGGGCCGGCTACTCTTCT<br>Reverse : AAGGAAGGCCCTCGAGAACA	(4) Purchased from Microsynth

Vectors, Drugs and Reagents

Item	Description / reference	supplier
pBApo-CMV Pur	Plasmid backbone in which secreted Gluc was cloned either downstream or upstream two HDV antigenomic ribozyme sequences	GenScript Biotech Corporation, Netherlands
pET-20b(+) () containing the HDV ribozyme sequence under a T7 promoter	Custom cloning	GenScript Biotech Corporation, Netherlands
Drug library APExBio	1971 approved drugs	Apexbt

Drug library Prestwick	1280 small molecules, 95% of which are approved drugs	Prestwick Chemical Libraries
Drug library Enamine	3393 compounds with antiviral activity	SIA Enamine
PC1-24781		MedChemExpress, USA
pracinostat		MedChemExpress, USA
entinostat		MedChemExpress, USA
8-azaguanine		MedChemExpress, USA
8-azaguanosine-5’-triphosphate	Custom synthesis	Jena Bioscience GmbH, Germany
In Vitro Toxicology Assay Kit	Ref : TOX1-1KT	Sigma-Aldrich
Restriction enzymes	BamH1, EcoR1, NaeI and HindIII	New England Biolabs
FastAP Thermosensitive Alkaline Phosphatase	Ref : EF0651	ThermoFischer Scientific
ReliaPrep™ DNA Clean-up and Concentration System	Ref : A2891	Promega
jetPRIME® transfection reagent	Ref : 114-15	Polyplus
Puromycin	Ref : ant-pr-1	Invivogen, Labforce
NucleoSpin Tissue DNA extraction kit	Ref : 740952.50	Macherey-Nagel
platinum Taq DNA polymerase	#M0267	New England Biolabs
Secrete-Pair™ Gaussia Luciferase Assay	Ref : LF062	Labomics
MEGAshortscriptTM in vitro transcription kit	Ref : AM1354	Invitrogen
NucleoSpin RNA II kit	Ref : 740955.50	Macherey-Nagel
Superscript II and random hexamer primers		Roche Diagnosis

Softwares

**Software name**	**Manufacturer**	**Version**
GraphPad Prism	Dotmatics	9.1.0

SnapGene	Dotmatics	6.1
Geneious	Dotmatics	10.0.7

Supplementary figures

Fig. S1

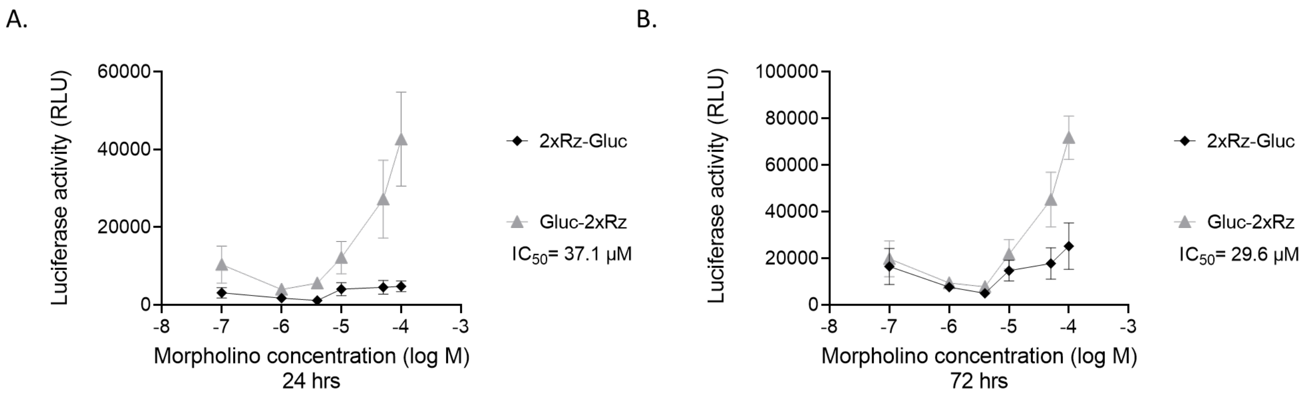

Fig. S1. Cell-based assay validation. Dose response experiments in 2xRz -Gluc and Gluc- 2xRz cells treated with increasing concentrations of antisense Morpholino at 24 hrs **(A)** or 72 hrs **(B)** post treatment. No effect in the luciferase induction was observed in 2xRz-Gluc transfected cells treated with the antisense Morpholino, whereas a dose mediated induction of the luciferase expression was detected in Gluc-2xRz transfected cells incubated with the antisense Morpholino (IC_{50}=37.1 µM at 24hrs and IC_{50}=29.6 µM at 72hrs).

Fig. S2

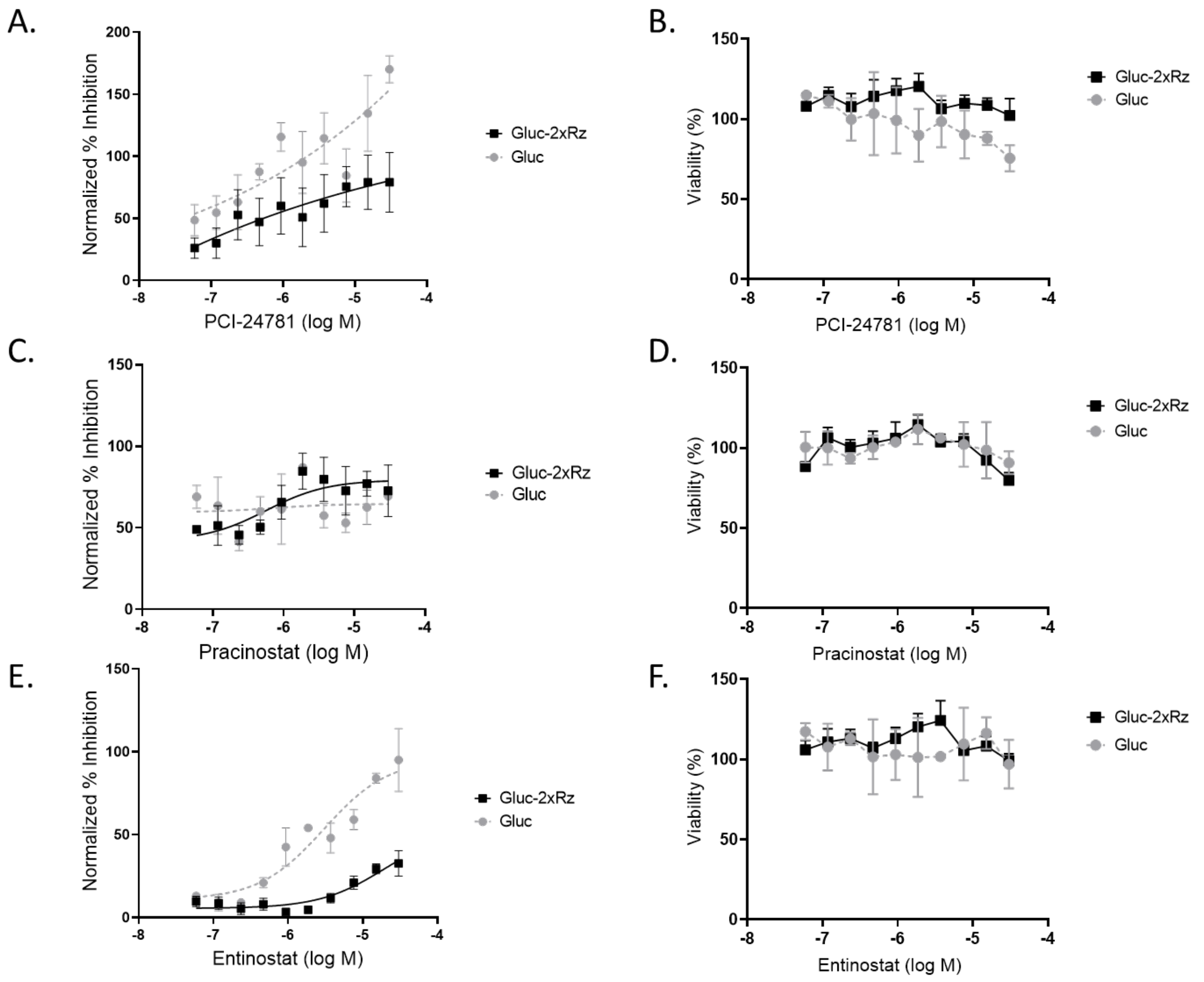

Fig. S2. Hit validation by performing dose response analysis. (A, C, E) Half-maximal inhibitory concentration (IC_{50}) of PC1-24781 (A), pracinostat (C) and entinostat (E) in Gluc-2xRz and Gluc cells. **(B, D, F)** Toxicity evaluation of PC1-24781 (B), pracinostat (D) and entinostat (F) by MTT assay.

Fig. S3

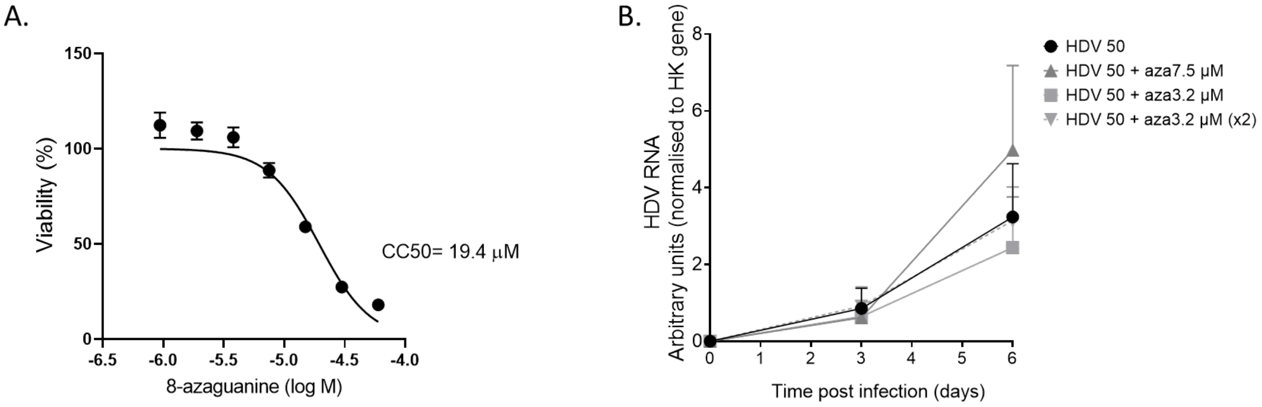

Fig. S3. Toxicity and antiviral effect of 8-azaguanine on HepNB2.7 cells. (A) Viability of HepNB2.7 cells treated with increasing concentration of 8-azaguanine was assessed at day 6 using the MTT assay. **(B)** HepNB2.7 cells were infected with 50 viral genome equivalents (vge)/cell of HDV and treated with 8-azaguanine at 3.2 µM and 7.5 µM 16 hrs post-infection. For the concentration of 3.2 µM, 8-azaguanine was added either once (16 hours post infection, grey solid line) or twice (second treatment at 3 days, grey dotted line). Levels of intracellular HDV RNA were assessed by RT-qPCR at 3- and 6-days post infection. Data are expressed as the mean ±SEM of at least 2 independent experiments.

Fig. S4

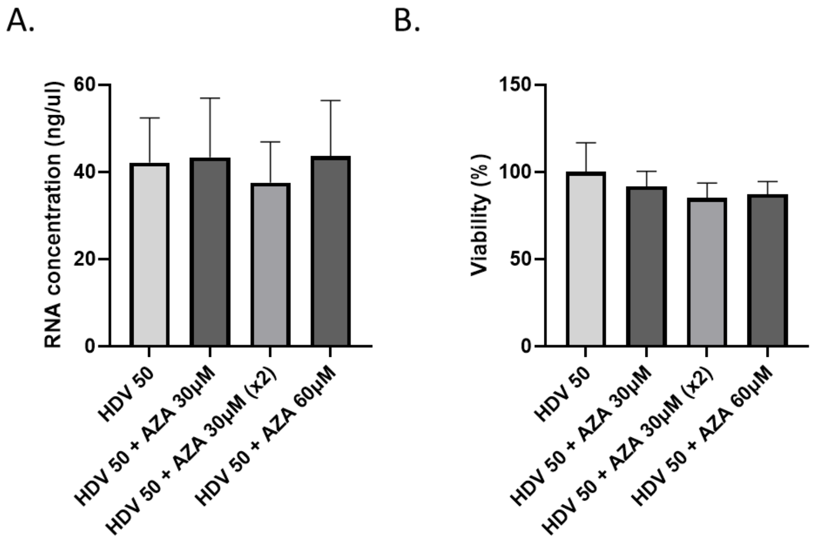

Fig. S4. Intracellular RNA levels and MTT assay on differentiated HepaRG cells. Differentiated HepaRG cells were infected with 50 vge/cell of HDV and treated with 8-azaguanine at 30 µM and 60 µM 16 hrs post-infection. For the concentration of 30 µM, 8-azaguanine was added either once (16 hours post infection, grey solid line) or twice (second treatment at 3 days, grey dotted line). **(A)** Intracellular RNA levels were assessed by RT-qPCR at 6-days post infection. **(B)** Viability of differentiated HepaRG cells treated with increasing concentration of 8-azaguanine was assessed at day 6 using the MTT assay. Data are expressed as the mean ±SEM of 3 independent experiments.

Supplementary references

1. Gripon P, Rumin S, Urban S, Le Seyec J, Glaise D, Cannie I, Guyomard C, et al. Infection of a human hepatoma cell line by hepatitis B virus. Proc Natl Acad Sci U S A 2002;99:15655-15660.
2. Alfaiate D, Lucifora J, Abeywickrama-Samarakoon N, Michelet M, Testoni B, Cortay JC, Sureau C, et al. HDV RNA replication is associated with HBV repression and interferon-stimulated genes induction in super-infected hepatocytes. Antiviral Res 2016;136:19-31.
3. Lempp FA, Schlund F, Rieble L, Nussbaum L, Link C, Zhang Z, Ni Y, et al. Recapitulation of HDV infection in a fully permissive hepatoma cell line allows efficient drug evaluation. Nat Commun 2019;10:2265.
4. Scholtes C, Icard V, Amiri M, Chevallier-Queyron P, Trabaud MA, Ramiere C, Zoulim F, et al. Standardized one-step real-time reverse transcription-PCR assay for universal detection and quantification of hepatitis delta virus from clinical samples in the presence of a heterologous internal-control RNA. J Clin Microbiol 2012;50:2126-2128.
