# Supplementary material for: Identification of selective hepatitis delta virus ribozyme inhibitors by high-throughput screening of small molecule libraries
Source: JHEP Rep. 2022 Dec 17;5(3):100652. doi: 10.1016/j.jhepr.2022.100652 (PMC9871325; doi:10.1016/j.jhepr.2022.100652)
Supplement: Multimedia component 2 [file mmc2.docx]

**Journal of Hepatology**

**CTAT methods**

Tables for a “Complete, Transparent, Accurate and Timely account” (CTAT) are now mandatory for all revised submissions. The aim is to enhance the reproducibility of methods.

- Only include the parts relevant to your study
- Refer to the CTAT in the main text as ‘Supplementary CTAT Table’
- Do not add subheadings
- Add as many rows as needed to include all information
- Only include one item per row

**If the CTAT form is not relevant to your study, please outline the reasons why:**

|  |
| --- |

- 1. **Antibodies**

| **Name** | **Citation** | **Supplier** | **Cat no.** | **Clone no.** |
| --- | --- | --- | --- | --- |
| **NA** |  |  |  |  |

- 1. **Cell lines**

| **Name** | **Citation** | **Supplier** | **Cat no.** | **Passage no.** | **Authentication test method** |
| --- | --- | --- | --- | --- | --- |
| HepaRG | (1) (2) | Provided by Julie Lucifora, INSERM, Lyon |  | Up to 20 |  |
| HepNB2.7 | (3) | Provided by Prof. Stephan Urban, University Hospital Heidelberg, Germany |  | Up to 20 |  |

- 1. **Organisms**

| **Name** | **Citation** | **Supplier** | **Strain** | **Sex** | **Age** | **Overall n number** |
| --- | --- | --- | --- | --- | --- | --- |
|  |  |  |  |  |  |  |

- 1. **Sequence based reagents**

| **Name** | **Sequence** | **Supplier** |
| --- | --- | --- |
| Morpholino | TGGCGATGCCATGCCGACCC | GeneTools (USA) |
| Primer CMV-IE | 5’CGCAAATGGGCGGTAGGCGTG3’ | Microsynth |
| Primer M13 | 5’TGTAAAACGACGGCCAGT3’ | Microsynth |
| Primers EEF1A1 | Fwd : AGCAAAAATGACCCACCAATG  Reverse : GGCCTGGATGGTTCAGGATA | Microsynth |
| Primers HDV | Fwd : CGGGCCGGCTACTCTTCT  Reverse : AAGGAAGGCCCTCGAGAACA | (4)  Purchased from Microsynth |

- 1. **Biological samples**

| **Description** | **Source** | **Identifier** |
| --- | --- | --- |
|  |  |  |

- 1. **Deposited data**

| **Name of repository** | **Identifier** | **Link** |
| --- | --- | --- |
|  |  |  |

- 1. **Software**

| **Software name** | **Manufacturer** | **Version** |
| --- | --- | --- |
| GraphPad Prism | Dotmatics | 9.1.0 |
| SnapGene | Dotmatics | 6.1 |
| Geneious | Dotmatics | 10.0.7 |

- 1. **Other (e.g. drugs, proteins, vectors etc.)**

| pBApo-CMV Pur | Plasmid backbone in which secreted Gluc was cloned either downstream or upstream two HDV antigenomic ribozyme sequences | GenScript Biotech Corporation, Netherlands |
| --- | --- | --- |
| pET-20b(+) () containing the HDV ribozyme sequence under a T7 promoter | Custom cloning | GenScript Biotech Corporation, Netherlands |
| Drug library APExBio | 1971 approved drugs | Apexbt |
| Drug library Prestwick | 1280 small molecules, 95% of which are approved drugs | Prestwick Chemical Libraries |
| Drug library Enamine | 3393 compounds with antiviral activity | SIA Enamine |
| PC1-24781 |  | MedChemExpress, USA |
| pracinostat |  | MedChemExpress, USA |
| entinostat |  | MedChemExpress, USA |
| 8-azaguanine |  | MedChemExpress, USA |
| 8-azaguanosine-5’-triphosphate | Custom synthesis | Jena Bioscience GmbH, Germany |
| In Vitro Toxicology Assay Kit | Ref : TOX1-1KT | Sigma-Aldrich |
| Restriction enzymes | BamH1, EcoR1, NaeI and HindIII | New England Biolabs |
| FastAP Thermosensitive Alkaline Phosphatase | Ref : EF0651 | ThermoFischer Scientific |
| ReliaPrep^TM^ DNA Clean-up and Concentration System | Ref : A2891 | Promega |
| jetPRIME® transfection reagent | Ref : 114-15 | Polyplus |
| Puromycin | Ref : ant-pr-1 | Invivogen, Labforce |
| NucleoSpin Tissue DNA extraction kit | Ref : 740952.50 | Macherey-Nagel |
| platinum Taq DNA polymerase | #M0267 | New England Biolabs |
| Secrete-Pair™ Gaussia Luciferase Assay | Ref : LF062 | Labomics |
| MEGAshortscriptTM in vitro transcription kit | Ref : AM1354 | Invitrogen |
| NucleoSpin RNA II kit | Ref : 740955.50 | Macherey-Nagel |
| Superscript II and random hexamer primers |  | Roche Diagnosis |

- 1. **Please provide the details of the corresponding methods author for the manuscript:**

| **Eirini Tseligka, UNIGE, 1 rue Michel Servet, 1211 Geneva, Switzerland** |
| --- |

**2.0 Please confirm for randomised controlled trials all versions of the clinical protocol are included in the submission. These will be published online as supplementary information.**

| **NA** |
| --- |

**REFERENCES**

1. Gripon P, Rumin S, Urban S, Le Seyec J, Glaise D, Cannie I, Guyomard C, et al. Infection of a human hepatoma cell line by hepatitis B virus. Proc Natl Acad Sci U S A 2002;99:15655-15660.

2. Alfaiate D, Lucifora J, Abeywickrama-Samarakoon N, Michelet M, Testoni B, Cortay JC, Sureau C, et al. HDV RNA replication is associated with HBV repression and interferon-stimulated genes induction in super-infected hepatocytes. Antiviral Res 2016;136:19-31.

3. Lempp FA, Schlund F, Rieble L, Nussbaum L, Link C, Zhang Z, Ni Y, et al. Recapitulation of HDV infection in a fully permissive hepatoma cell line allows efficient drug evaluation. Nat Commun 2019;10:2265.

4. Scholtes C, Icard V, Amiri M, Chevallier-Queyron P, Trabaud MA, Ramiere C, Zoulim F, et al. Standardized one-step real-time reverse transcription-PCR assay for universal detection and quantification of hepatitis delta virus from clinical samples in the presence of a heterologous internal-control RNA. J Clin Microbiol 2012;50:2126-2128.
